# Trends in Overall Survival in Lung Adenocarcinoma with EGFR Mutation, KRAS Mutation, or No Mutation

**DOI:** 10.3390/cancers17071237

**Published:** 2025-04-05

**Authors:** Martin Faehling, Sabine Fallscheer, Birgit Schwenk, Harald Seifarth, Jörn Sträter, Claudia Lengerke, Petros Christopoulos

**Affiliations:** 1 Clinic of Cardiology and Pneumology, Esslingen Hospital, 73730 Esslingen, Germany; 2 Clinic of Radiology, University of Münster, 48143 Münster, Germany; 3 Clinic of Radiology, Esslingen Hospital, 73730 Esslingen, Germany; 4 Institute of Pathology, 73730 Esslingen, Germany; 5 University Hospital Tübingen, Department for Internal Medicine II, University of Tübingen, 72076 Tübingen, Germany; 6 German Cancer Consortium (DKTK), Partner Site Tübingen, a Partnership Between DKFZ and University Hospital Tübingen, 72076 Tübingen, Germany; 7 Department of Medical Oncology, Thorax Clinic, 69126 Heidelberg, Germany; 8 National Center for Tumor Diseases, Heidelberg University Hospital, 69126 Heidelberg, Germany

**Keywords:** *KRAS*, G12D, *EGFR*, Exon 21, lung cancer, check point inhibitor, real-world data, overall survival

## Abstract

Background: Treatment of lung adenocarcinoma has changed and now includes checkpoint inhibitors (CPIs) or, in the case of an *EGFR* mutation, third-generation EGFR TKI osimertinib. Few data compare the long-term overall survival (OS) of current and historic subgroups. Methods: This real-world analysis (KOMPASS study) included stage IV lung-adenocarcinoma patients with either EGFR, KRAS, or no mutation. Patients were assigned to the “current” *EGFR*, *KRAS*, or no-mutation cohort if they had mutation testing using NGS (*n* = 199; median date of diagnosis 2021). If they had an EGFR PCR test only, they were assigned to the “historic” *EGFR* or no-mutation cohort (*n* = 127; median date of diagnosis 2014). Results: Both the current and the historic *EGFR* cohorts had significantly longer OS than the respective no-mutation cohorts (HR 0.58 and 0.60, respectively). The current no-mutation and *EGFR* cohorts had a strong trend to longer OS than the respective historic cohorts. In the no-mutation cohorts, the improvement was due to an increase in long-term survivors (HR 0.71), whereas in the *EGFR* mutation cohorts, the median OS was improved without long-term survivors (HR 0.70). The *KRAS* cohort showed OS like the no-mutation cohort, with a plateau of long-term survivors around 20%. Conclusions: A comparison of our data with that of the phase III trials KEYNOTE-189 and FLAURA suggests that the improved outcomes are due to the use of CPIs or osimertinib. The clinical trial results are well translated into real-world clinical practice with comparable OS. *KRAS* patients benefit from CPI treatment like no-mutation patients.

## Simple Summary

This study compares the long-term survival of patients with stage IV lung adenocarcinoma treated with current therapies (checkpoint inhibitors or osimertinib for *EGFR* mutations) versus older treatments. The analysis included patients with *EGFR*, *KRAS*, or no mutations, divided into “current” and “historic” cohorts based on the timing of their mutation testing. Results showed that both current *EGFR* and no-mutation cohorts had significantly longer overall survival (OS) compared to historic cohorts. The improvement in survival was particularly notable for long-term survivors in the no-mutation group. *KRAS* patients had survival outcomes similar to those with no mutations, with about 20% reaching long-term survival. Overall, the findings suggest that newer treatments like checkpoint inhibitors and osimertinib have led to improved outcomes, aligning with phase III trial results and demonstrating their effectiveness in real-world practice.

## 1. Introduction

Lung cancer is the second most common cause of cancer and remains the leading cause of cancer death [1,2]. Non-small cell lung cancer (NSCLC) accounts for 81% of lung cancers, with most patients presenting in advanced stages [3,4,5]. NSCLC, in particular lung adenocarcinoma, is characterized by molecularly defined subsets with targetable oncogenic drivers. In lung adenocarcinoma, mutations of the *EGFR* or *KRAS* gene represent the most common oncogenic drivers [6]. Common *EGFR* mutations (del exon 19 or exon 21 L858R) are associated with better overall survival (OS) compared to no-mutation lung adenocarcinoma [7,8]. *KRAS* mutations were found to be a negative prognostic parameter in NSCLC [7,8,9], whereas other studies did not find a difference in outcome after chemotherapy [10,11]. However, these data come from a time during which immunotherapy with checkpoint inhibitors (CPIs) was not widely available. The regular use of CPIs has been shown to be associated with improved OS in no-driver patients and *KRAS* patients but not in *EGFR* patients [12,13]. Treatment with the third-generation EGFR tyrosine-kinase inhibitor (TKI) osimertinib results in improved OS in *EGFR* del exon 19 patients but not in *EGFR* exon 21 L858R patients [14]. The regular use of CPIs or osimertinib may have changed the prognostic implications of *EGFR* or *KRAS* mutations compared to no-driver patients. Therefore, we provide data on trends in OS of unselected patients with stage IV lung adenocarcinoma and *EGFR* or *KRAS* mutation, or no-mutation in a current and historic real-world population.

Furthermore, data from randomized clinical trials of targeted therapies for *EGFR* or *KRAS* mutant NSCLC concentrate on patients with classical *EGFR* mutations or with *KRAS* G12C mutation. Following a pooled post-hoc analysis of the LUX-Lung trials and a more recent report, uncommon *EGFR* mutations are still generally treated with the second-generation *EGFR* TKI afatinib and are associated with poor survival [15,16,17]. Only recently, a retrospective analysis showed responsiveness to osimertinib [18]. Taken together, there is still a lack of data on the current survival of patients with uncommon *EGFR* mutations. Similarly, there are almost no data on the survival of patients with specific *KRAS*-nonG12C mutations [19]. However, in light of the development of pan-RAS inhibitors, which are currently reaching the early clinical trial stage [20,21], data on the prognostic significance of specific *KRAS*-nonG12C mutations are of special interest. To close these gaps, we provide data on the survival of patients with uncommon *EGFR* mutations and preliminary data on the survival of patients with various *KRAS*-nonG12C subtypes.

## 2. Materials and Methods

This retrospective real-world analysis was conducted as part of the KOMPASS study at an experienced lung cancer center certified by the German Cancer Society (DKG). Patients diagnosed with NSCLC and adenocarcinoma histology until 30 September 2024 were eligible if molecular pathology results for *EGFR* and *ALK* were available. All patients had histologically confirmed NSCLC with adenocarcinoma histology in the diagnostic biopsy, and complete tumor staging, including PET CT if clinically indicated, and contrast-enhanced MRI of the brain or, if MRI was contraindicated, contrast-enhanced CT at baseline. Staging was performed according to the IASLC 8th edition [22]. In earlier cases, molecular pathology was performed using (multiplex) polymerase-chain reaction (PCR) for *EGFR* Exon 18, 19, 20, and 21, and fluorescence in situ hybridization (FISH) for *ALK* translocations, not including *KRAS* testing. Between 2016 and 2020, molecular testing was gradually changed to DNA- and RNA-based next-generation sequencing (NGS) from tissue or peripheral blood (liquid biopsy) targeting a lung cancer panel. Patients who had an NGS test result available were assigned to the “current” cohort. Patients with a PCR result only were assigned to the “historic” cohort. Predictive *EGFR* mutations were grouped in exon 19 deletions, exon 21 L858R, and “uncommon” mutations (exon 18, exon 21 non-L858R, or complex mutations). For comparison with phase III clinical trial populations (KEYNOTE-189, FLAURA) [14,23], current and historic no-mutation* and EGFR* subpopulations were analyzed comprising only patients with good performance scores (ECOG 0-1) and common *EGFR* mutations (del exon 19 or exon 21 L858R).

Treatment was performed in accordance with national and international guidelines (Onkopedia [24], ESMO [25,26]). Each patient was discussed at least once in the multidisciplinary tumor board (MDT). If deemed necessary by the MDT, patients were additionally presented to the molecular tumor board (University of Heidelberg, Germany). The study was performed in accordance with the International Conference on Harmonization Guidelines on Good Clinical Practice and the Declaration of Helsinki. The KOMPASS study was approved by the ethics committee of Landesärztekammer Baden-Württemberg (F-2017-004, F-2019-092). Patients provided written informed consent. There was no funding.

The databank was locked on 28 February 2025. The original data will be made available as a data paper. For clarity and simplicity, some Kaplan–Meier plots contain more than two curves. For survival analysis, we performed Cox proportional hazard regression on pairwise Kaplan–Meier plots. Hazard ratios (HR), 95% confidence intervals (CI), and *p*-values were calculated using the log-rank (Mantel–Cox) test. Assuming non-parametric data, the significance of numeric values were calculated using the Mann–Whitney U-test. Kaplan–Meier plots and all statistical analyses were generated using GraphPad Prism8. The report is in line with the STROBE statement [27].

## 3. Results

### 3.1. Structure of Study Population

**Figure 1:**
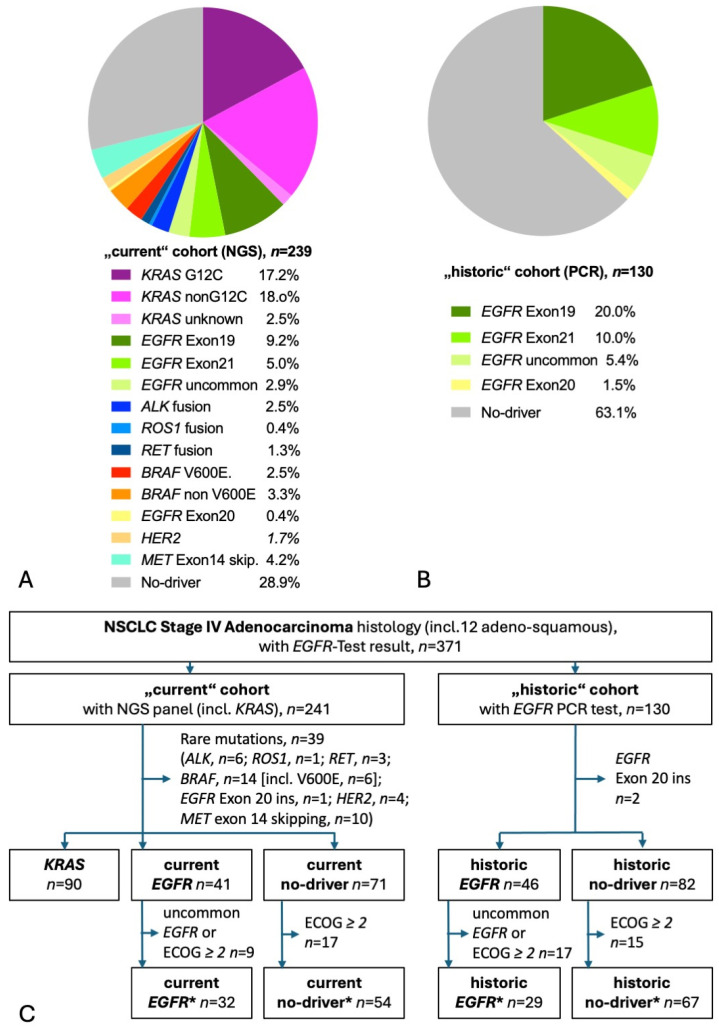
Detected mutations and patient flow. (**A**) Pie diagram of patients with NGS molecular test results. Of the 238 NGS test results, 192 had used tissue only, 10 had NGS on liquid biopsy (blood) only, and 36 had NGS both on tissue and liquid biopsy. (**B**) Pie diagram of patients with EGFR-PCR test results. (**C**) CONSORT diagram. The “star” (*) cohorts contain only patients fulfilling the major inclusion criteria of the respective phase III studies KEYNOTE-189 and FLAURA.

A total of 367 patients had molecular test results available, of those, 238 had used NGS (“current” cohort), and 129 had PCR results (“historic” cohort). Figure 1A shows the distribution of detected mutations in the current cohort. *KRAS* mutations were the most common mutations found in more than a third of tested patients. Almost half of *KRAS* patients had the targetable G12C mutation. The second most common were EGFR mutations, found in 17%. Rare mutations were found in every sixth patient and no-driver in almost 30%. Figure 1B shows the distribution of detected mutations in the historic cohort, with an *EGFR* mutation in exon 19, 21, or 18 detected in one-third of tested patients. Taking both current and historic *EGFR* patients together, 55% harbored the prognostically beneficial del exon 19 mutation, 29% showed the *EGFR* exon 21 L858R mutation, and 16% had an uncommon mutation [14,16,18]. The CONSORT diagram shows the selection process of patients for this analysis (Figure 1C) [28]. A total of 199 current patients had adenocarcinoma stage IV with either *KRAS* mutation (*n* = 90), *EGFR* mutation (*n* = 41), or no known driver (*n* = 68). A total of 127 historic patients had adenocarcinoma stage IV with either *EGFR* mutation (*n* = 46) or no known driver (*n* = 81). Table 1 gives baseline characteristics and survival. At the time of the database lock, there were 132 deaths (66%), with a median follow-up of living patients of 33.3 months in the current cohort and 117 deaths (92%) with a median follow-up of 87.8 months in the historic cohort. In the current cohort, one-quarter of the living patients had a follow-up of more than 5 years. In comparison with a general NSCLC population, the patients of both the current and historic cohorts had a high proportion of female patients and of never-smokers. As expected in a population enriched in women and non-smokers, the proportion of TTF-1-positive adenocarcinoma was high [29]. In line with the fourfold higher likelihood of an *EGFR* mutation in TTF1-positive adenocarcinoma, almost every *EGFR* mutation-positive histology was TTF1-positive adenocarcinoma [30]. A PD-L1 TPS was available for 86% of the current cohort and for 37% of the historic cohort. In line with previous reports, PD-L1 TPS was significantly higher in *KRAS* patients and no-driver patients compared to *EGFR* patients [31,32,33,34]. The most common treatment of *EGFR* patients was an EGFR TKI (100% and 91% in the current and historic cohort, respectively). Of note, only 22% of historic *EGFR* patients received a third-generation EGFR TKI (osimertinib) as opposed to 88% of current *EGFR* patients. The most common treatment of *KRAS* patients and no-driver patients was a CPI (88% and 76%, respectively). Since the historic no-driver patients were diagnosed at a time around 2014, when first-line CPIs were not approved, the historic cohort had little and, if any, later-line CPI treatment.

The historic *EGFR* and no-driver cohorts were diagnosed at a similar time (median date of diagnosis 2014) and were diagnosed about 7 years earlier than the respective current cohorts (median date of diagnosis 2021). The current cohorts had a higher use of PET-CT for staging and a slightly higher proportion of stage IVA patients. Otherwise, the baseline characteristics were well-balanced. These considerations hold true for the no-mutation* cohorts and the EGFR* cohorts as well (Table S1). A high proportion of the current no-mutation* cohort received first-line CPIs (64%, most commonly pembrolizumab [60%]), whereas only a minority of the historic no-mutation* cohort received first-line CPIs (5%). Thus, the current and historic no-mutation* cohorts match with the arms of the KEYNOTE-189 trial [23]. All EGFR* patients received first-line EGFR TKI. For the current EGFR* cohort, this was mainly osimertinib (94%), and for the historic EGFR* cohort, this was mainly a first-generation EGFR TKI (79%). Thus, the current and historic EGFR* cohorts match well with the arms of the FLAURA trial [14].

### 3.2. Analysis of Overall Survival

#### 3.2.1. Gene-Wise Analysis of Overall Survival

**Figure 2:**
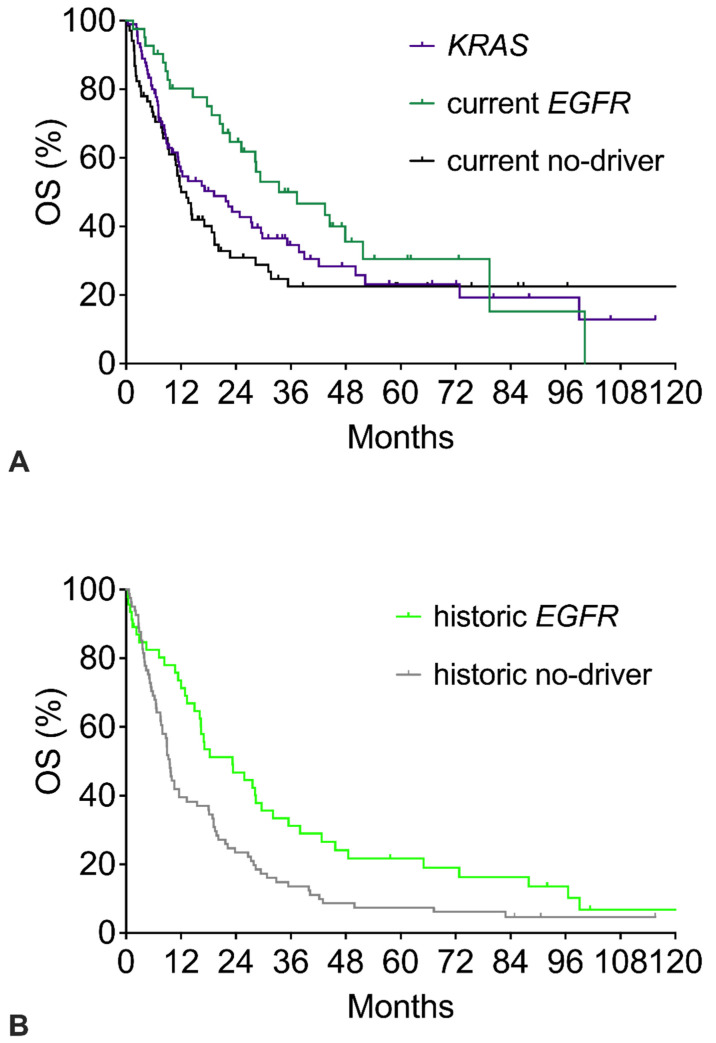
Overall survival (OS) of patients with specific mutations: *EGFR* mutation, *KRAS* mutation, or no-driver patients. (**A**) OS of current no-driver patients, current *EGFR* patients, and *KRAS* patients. (**B**) OS of historic no-driver patients and historic *EGFR* patients. The corresponding numerical values and statistics are given in Table 2.

Figure 2 shows the survival of the three current cohorts (Figure 2A) and the two historic cohorts (Figure 2B). Compared to the respective no-driver cohorts, both the current and the historic *EGFR* cohorts had significantly better OS with similar hazard ratios around 0.59 (Table 2). However, beyond 6 years, the tail of the current *EGFR* Kaplan–Meier survival curve shows a late further decline, whereas the current no-driver curve remains stable at a plateau of 22%. In the historic cohorts, the tail of the current *EGFR* survival curve again shows a late further decline. The historic no-driver curve continuously declines to about 5% and does not show a plateau. The *KRAS* curve shows a course like the current no-driver cohort with a plateau of 20% long-term survivors (Figure 2A). OS showed a minor trend in favor of *KRAS* patients (HR 0.841 for *KRAS* vs. no-driver).

#### 3.2.2. Trends in Overall Survival

**Figure 3:**
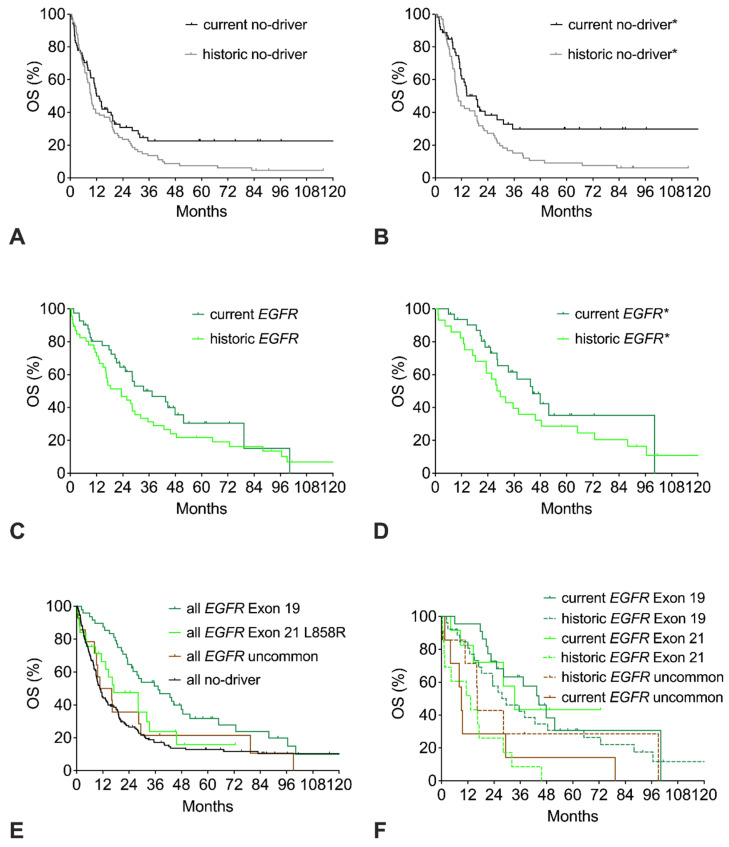
Trends in overall survival (OS) of no-driver patients, *EGFR* patients, or *EGFR* mutation subtypes. (**A**) OS of current or historic no-driver patients. (**B**) OS of current or historic no-driver* patients (includes only ECOG 0-1 patients). (**C**) OS of current or historic *EGFR* patients. (**D**) OS of current or historic EGFR* patients (includes only patients with common *EGFR* mutations and ECOG 0-1). (**E**) OS of all *EGFR* patients with del Exon 19 mutation, Exon 21 L858R mutation, uncommon *EGFR* mutation, or no-driver patients (includes both current and historic cohorts). (**F**) OS of current and historic *EGFR* patients with del Exon 19 mutation, Exon 21 L858R mutation, or uncommon *EGFR* mutation.

The survival curves of the current and the historic no-driver cohorts show a similar early decline with only 40% of patients alive at 18 months. However, beyond 18 months, the curves clearly separate, resulting in a strong trend to improved OS of the current cohort with a HR of 0.71 (*p* = 0.06; Figure 3A, Table 2). Restricting the analysis to good-performance patients does not lead to a relevant change in the historic cohort but results in an improvement of the current no-driver* cohort, with an elevation of the long-term plateau from 25% to 31% resulting in significantly improved OS with a HR of 0.60 (Figure 3B).

#### 3.2.3. Overall Survival of *EGFR*-Mutation Subtypes

Compared to the historic *EGFR* cohort, the current *EGFR* cohort has a trend to improved OS (Figure 3C, Table 2). Restricting the analysis of the *EGFR* cohorts to patients with common mutations and good performance score (ECOG 0-1) results in about six months longer median OS with a similar trend to improved OS in favor of the current cohort (EGFR* cohorts, Figure 3D). With respect to the specific mutation subtype, the *EGFR* del exon 19 cohort had significantly longer OS than the *EGFR* exon 21 L858R cohort or the uncommon *EGFR* cohort (Figure 3E). Compared to the no-driver cohort, only the *EGFR* exon 19 cohort had significantly longer OS. The *EGFR* exon 21 L858R cohort had a trend to improved survival, whereas the uncommon *EGFR* cohort had similar OS compared to the no-driver cohort. Both the current *EGFR* del exon 19 cohort and the current *EGFR* exon 21 cohort had improved survival compared to the respective historic cohorts (Figure 3F). In contrast, no improvement was seen for the uncommon *EGFR* cohorts.

#### 3.2.4. Overall Survival of *KRAS*-Mutation Subtypes

**Figure 4:**
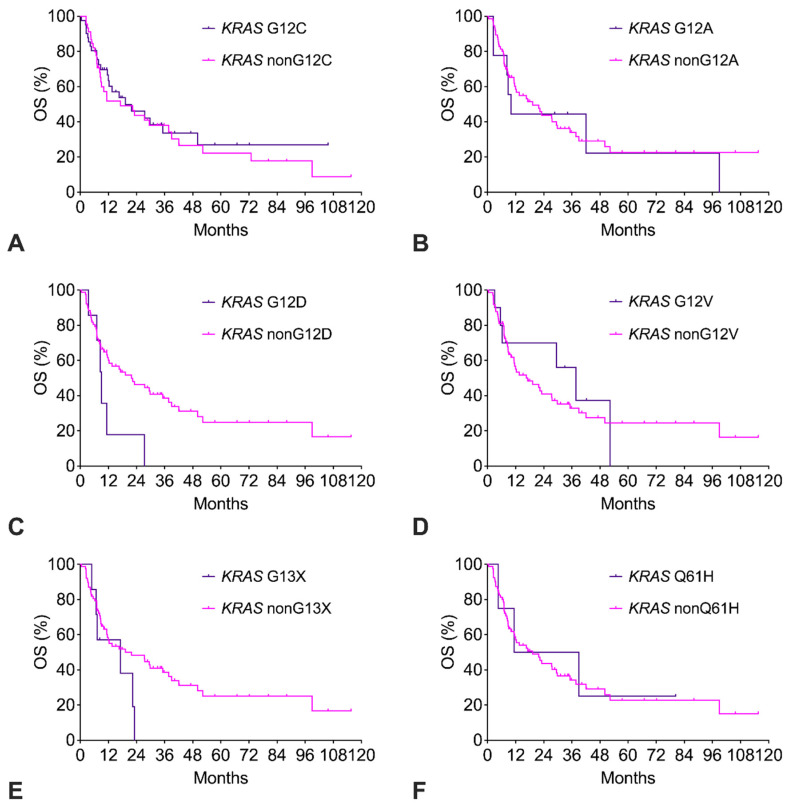
(**A**–**F**). Overall survival (OS) of patients with *KRAS* mutation subtypes.

The *KRAS* G12C cohort had similar OS compared to the *KRAS* nonG12C cohort (Figure 4A). Of note, only 14% of *KRAS* G12C patients had received a G12C-targeted therapy (sotorasib, adagrasib). The exploratory survival analysis of the other *KRAS* subtypes with *n* ≥ 4 is shown in Figure 4B–F. For *KRAS* G12A, G12V, and Q61H, OS was like the remaining *KRAS* subtypes. In contrast, for *KRAS* G12D and *KRAS* G13X, there was a strong trend to impaired OS with a more than twofold increased risk of death (Table 2).

Subtypes with *n* ≥ 4 were analyzed. The corresponding numerical values and statistics are given in Table S2.

## 4. Discussion

The high proportion of females and never-smokers as well as the low PD-L1 TPS in no-driver patients implies a selection by the treating physician towards testing preferentially patients with a high likelihood of a driver mutation. This accounts for the high proportion of patients with a targetable driver mutation in our cohort exceeding the proportion of 50% to 66% generally reported for Western adenocarcinoma populations [35,36,37,38,39,40]. The distribution of detected mutations in our cohort is in line with these reports.

Compared to the respective historic cohorts, both the current *EGFR* cohort and the current no-driver cohort show clinically relevant improvements in OS with a different pattern of change in the Kaplan–Meier curves. The current *EGFR* cohort has a marked improvement in median OS with little change in long-term survival, whereas the no-driver cohort experiences little change in median OS but a marked improvement in long-term survival. Since these changes in OS occurred without a change in the lung cancer center setting, they imply an effect of different treatment changes on OS.

More than three-quarters of current no-driver patients received immuno-oncological therapy with CPIs, most commonly first line. In the historic cohort, only 5% received first-line CPIs, and 70% did not receive any CPI. The no-driver* cohorts comprised patients fulfilling the main inclusion criteria of the KEYNOTE-189 phase III trial comparing the pembrolizumab plus chemotherapy with chemotherapy alone [23]. OS of the current no-driver* cohort with a tail of 30% long-term survivors is comparable with OS of the KEYNOTE-189 pembrolizumab arm, whereas OS of the historic no-driver* resembles OS of the chemotherapy arm with no long-term survivors (Supplemental Table S3A) [41]. Taken together, the improved OS of current no-driver patients is likely to reflect the benefit of immuno-oncological therapy with CPIs [42]. Conversely, the lack of long-term survivors despite CPI therapy in every fourth current *EGFR*-mutation-positive patient is in line with the literature that these patients do not derive a long-term benefit from immuno-oncological therapy with CPIs [43].

The EGFR* cohorts comprised patients fulfilling the main inclusion criteria of the FLAURA phase III trial comparing the third-generation EGFR TKI osimertinib with the first-generation *EGFR* TKIs erlotinib or gefitinib (“comparator”) [14,44,45]. Like the FLAURA-verum patients, the current EGFR* patients mainly received first-line osimertinib, whereas most historic EGFR* patients received a first-line, first-generation EGFR TKI (Supplemental Table S3B). OS of the current EGFR* cohort is comparable with OS of the FLAURA osimertinib arm, whereas OS of the historic EGFR* resembles OS of the comparator arm. Our data suggest that the improvement in OS of *EGFR*-mutant patients is due to the switch of treatment from first-generation EGFR TKIs to third-generation EGFR TKI osimertinib following publication of the FLAURA trial. In contrast to a recent French and British real-world analysis, we find that real-world OS is at least comparable to OS in the FLAURA trial [46,47].

With respect to *EGFR*-mutation subtypes, our data provide another confirmation of the prognostically beneficial effect of the *EGFR* del Exon 19 mutation compared to other *EGFR* mutations in a real-world population with regular access to EGFR-TKI treatment [14,16,18]. The current cohorts of both common *EGFR*-mutation subtypes had improved OS compared to the respective historic cohorts. Since in both common *EGFR*-mutation subtypes, the mainstay of treatment was a first-generation EGFR TKI in the historic cohorts and changed to osimertinib in the current cohorts, our data suggest that both *EGFR* del exon 19 patients and *EGFR* exon 21 L858R patients benefit from osimertinib in terms of OS. In contrast, the FLAURA trial showed an OS benefit only for *EGFR* del exon 19 patients but not for *EGFR* exon 21 L858R patients; however, both *EGFR* subtypes had a benefit in progression-free survival [14,44]. A possible explanation may be that the improved tolerability of osimertinib compared to first-generation EGFR-TKIs leads to better compliance in the real-world setting.

In contrast to most trials on EGFR-TKIs, our EGFR cohort includes 15% of patients with an uncommon *EGFR* mutation. In line with the literature, these patients had worse OS compared to the common *EGFR* exon 19 and exon 21 mutations [15,48,49,50,51]. No improvement in survival was found in the uncommon *EGFR* cohorts. No change in treatment was observed with first- and second-generation EGFR TKIs remaining the mainstay of treatment for patients with uncommon *EGFR* mutations. This highlights the need for further research to improve the outcome in patients with uncommon *EGFR* mutations, which were found in about 3% of our population, slightly exceeding the frequency of *ALK* fusions with many specific targeted treatments available. Taken together, our data on all *EGFR* cohorts emphasize the need for the development of treatments focusing on long-term tumor control, e.g., by immune-combination strategies, which might make *EGFR*-tumor cells more “visible” to the immune system.

In line with previous registry reports, our data show high levels of PD-L1 expression in the *KRAS* cohort [34]. The survival curve of our *KRAS* cohort resembles that of the no-driver patients. In line with another report of a recent *KRAS* cohort with a documented high proportion of treatment with CPIs [52], our data do not support the previously reported negative prognostic effect of *KRAS* on OS [7,8,9]. Since our *KRAS* cohort has received a high rate of CPI treatment and since the survival curve shows a relevant tail of long-term survivors similar to the phase III CPI trials [41,53,54], our data suggest that patients with *KRAS* mutations generally benefit from CPI treatment. However, in line with our findings and a recent real-world report [55], *KRAS* G12D patients did not benefit from CPIs in the POSEIDON trial [56]. Our data suggest that *KRAS* G13X patients may also not respond to CPIs, but there are no corresponding reports in the literature. Taken together, our data on the *KRAS* cohort emphasize the need for further development of both *KRAS* G12C inhibitors and pan-RAS inhibitors into first-line therapy, ideally combined with first-line immunotherapy to achieve better medium OS driven by the *KRAS*-targeted treatment and better long-term OS driven by immunotherapy.

The limitations of our study include the intrinsic heterogeneity of patients, limited patient numbers in subgroups, and the variable follow-up period. Strengths of our study include a mature dataset of a current lung cancer population with homogeneous detailed clinical characterization, staging, and treatment in an expert lung cancer referral center, with the hard endpoint of OS and survival data beyond 5 years of follow-up. Our real-world data offer the opportunity to compare different patient populations in the same healthcare setting, which would require cross-trial comparisons of randomized clinical trials.

## 5. Conclusions

Our real-world study shows that OS in no-mutation patients and *EGFR* patients with common mutations improved to a similar extent as in the respective phase III clinical trials, likely due to the use of CPIs or osimertinib, respectively. Except for G12D and possibly G13X patients, *KRAS* patients benefit from CPIs as well.

## Figures and Tables

**Table 1:** Baseline characteristics, treatment, and follow-up of the current and historic cohorts.

	Current Cohort (NGS), *n* = 199			Historic Cohort (PCR), *n* = 127	
	*KRAS*	Current *EGFR*	Current No-Driver	Historic *EGFR*	Historic No-Driver
Number	90	41	68	46	81
Date of diagnosis (median, IQR)	8/2020 3/18 to 10/22	3/2021 8/19 to 8/22	4/2021 2/18 to 3/23	11/2013 6/11 to 1/17	5/2014 8/12 to 5/16
Age (median, range)	65.1 (39.1–86.8)	68.4 (37.4–87.2)	68.9 (32.3–90.8)	71.8 (38.8–89.6)	66.5 (44.6–84.8)
**Sex**					
Male	46 (51%)	10 (24%)	41 (60%)	14 (30%)	42 (52%)
Female	44 (49%)	31 (76%)	27 (40%)	32 (70%)	39 (48%)
**Performance** **status**					
ECOG 0	25 (28%)	22 (54%)	12 (18%)	13 (28%)	18 (22%)
ECOG 1	54 (60%)	15 (37%)	41 (60%)	21 (46%)	48 (59%)
ECOG 2	9 (10%)	4 (10%)	10 (15%)	10 (22%)	12 (15%)
ECOG 3	2 (2%)	0	5 (7%)	2 (4%)	3 (4%)
**Smoking status**	NA 1		NA 2		NA 1
Never-smoker	5 (6%)	19 (46%)	6 (9%)	27 (59%)	16 (20%)
Ever smoker	84 (94%)	22 (54%)	60 (91%)	19 (41%)	64 (80%)
Pack Years (median) ^a^	*40*	*12*	*40*	*20*	*40*
**Histology**					
Adenocarcinoma	87 (97%)	38 (93%)	68 (100%)	46 (100%)	80 (99%)
Adeno-squamous carcinoma	3 (3%)	3 (7%)	0	0	1 (1%)
TTF1-pos.	*64 (80%)*	*35 (100%)*	*52 (88%)*	*26 (96%)*	38 (68%)
TTF1-neg.	*16 (20%)*	*0*	*7 (12%)*	*1 (4%)*	18 (32%)
TTF1 NA	*10*	*6*	*9*	*19*	*25*
**PD-L1 (TPS)** ^b^	NA 12	NA 6	NA 9	NA 34	NA 46
0%	23 (29%)	23 (66%)	28 (48%)	5 (42%)	22 (63%)
1–49%	23 (29%)	9 (26%)	17 (29%)	4 (33%)	8 (23%)
50–100%	32 (41%)	3 (9%)	14 (24%)	3 (25%)	5 (14%)
**Stage (UICC 8)**					
Staging included PET-CT	63 (70%)	22 (54%)	39 (57%)	9 (20%)	24 (30%)
IVA	36 (40%)	15 (37%)	30 (44%)	14 (30%)	23 (28%)
IVB	54 (60%)	26 (63%)	38 (56%)	32 (70%)	58 (72%)
**Treatment** **included**			NA 1		
Targeted therapy ^c^	6 (14%)	41 (100%)	0	42 (91%)	0
*Osimertinib 1L* ^d^	-	*32 (78%)*	-	*5 (11%)*	-
*Osimertinib ≥ 2L*	-	*4 (10%)*	-	*5 (11%)*	-
CPI	79 (88%)	11 (27%)	53 (78%)	2 (4%)	24 (30%)
*CPI 1L*	*67 (74%)*	*1 (2%)*	*40 (59%)*	*1 (2%)*	*5 (6%)*
*CPI ≥ 2L*	*12 (13%)*	*10 (24%)*	*13 (19%)*	*1 (2%)*	*19 (23%)*
BSC only	4 (4%)	0	6 (9%)	3 (7%)	3 (4%)
Follow-up of living patients (months) [median (range)]	23.3 5.1–115.6	44.7 6.9–72.7	36.0 3.8–144.3	74.9 1.6–151.8	87.8 67.4–115.6
**Deaths**	58 (64%)	26 (63%)	48 (71%)	40 (87%)	77 (95%)
Death due to lung cancer	*49 (54%)*	*24 (59%)*	*34 (50%)*	*31 (67%)* ^e^	*72(89%)*
Death due to other causes	*9 (10%)*	*2 (5%)*	*14 (21%)*	*8 (17%)*	*5 (6%)*

CPI: checkpoint inhibitor; IQR: inter-quartile range; NA: not assessed; NGS: next-generation sequencing; TKI: tyrosine-kinase inhibitor; TPS: tumor proportion score. The cohorts were compared using the Mann–Whitney U-test. ^a^ *EGFR* patients had smoked significantly fewer pack years (PY) than *KRAS* patients (*p* = 0.0003) or no-driver patients (mean 45.9 PY, *p* < 0.0001). ^b^ *KRAS* patients had significantly higher PD-L1 TPS (mean 37.9%) than current *EGFR* patients (mean 11.0%, *p* < 0.0001) or current no-driver patients (mean 20.4%, *p* = 0.0003). Current no-driver patients had significantly higher PD-L1 TPS than current *EGFR* patients (*p* = 0.020). ^c^ Targeted therapy: For *KRAS* G12C-positive patients, KRAS-inhibitors, for *EGFR* patients, EGFR-TKI; ^d^ 91% of current *EGFR* del exon 19 patients received 1 L osimertinib, 100% of current *EGFR* exon 21 L858R patients received 1 L osimertinib, 14% of current uncommon *EGFR* patients received 1L osimertinib, 15% of historic *EGFR* del exon 19 patients received 1 L osimertinib, 8% of historic *EGFR* exon 21 L858R patients received 1 L osimertinib. None of the historic uncommon *EGFR* patients received 1 L osimertinib. ^e^ One cause of death was unknown.

**Table 2:** Summary of survival data in subgroups.

Parameter	Overall Survival (Months)		Comparison Between Subgroups		
	*n*	Median	HR	CI	*p*
**Current**	**(Figure 2A)**				
current *EGFR*	41	37.3	0.581 (vs. current no-driver)	0.366–0.922	0.021 *
			0.700 (vs. *KRAS*)	0.450–1.09	0.114
*KRAS*	90	19.2	0.841 (vs. current no-driver)	0.571–1.24	0.382
current no-driver	68	12.0	-	-	-
**Historic**	**(Figure 2B)**				
historic *EGFR*	46	23.3	0.598 (vs. no-driver)	0.413–0.865	0.0063 **
historic no-driver	81	9.5	-	-	-
**Trends**	**(Figure 3A–D)**				
current no-driver	68	12.0	0.713 (vs. historic no-driver)	0.501–1.01	0.060
historic no-driver	81	9.5	-	-	-
current no-driver *	53	18.7	0.597 (vs. historic no-driver *)	0.399–0.894	0.012 *
historic no-driver *	66	10.3	-	-	-
current *EGFR*	41	37.3	0.699 (vs. historic *EGFR*)	0.428–1.14	0.152
historic *EGFR*	46	23.3	-	-	-
current *EGFR* *	32	44.5	0.684 (vs. historic *EGFR* *)	0.365–1.28	0.236
historic *EGFR* *	29	29.6	-	-	-
***EGFR* subtypes**	**(Figure 3E–F)**				
all *EGFR* del exon 19	48	38.0	0.475 (vs. all exon 21)	0.240–0.939	0.032 *
			0.404 (vs. all uncommon)	0.184–0.889	0.024 *
			0.510 (vs. all no-driver)	0.369–0.706	<0.0001 ***
all *EGFR* exon 21 L858R	25	17.1	0.886 (vs. uncommon)	0.410–1.92	0.759
			0.752 (vs. all no-driver)	0.478–1.18	0.217
all *EGFR* uncommon	14	13.4	0.939 (vs. all no-driver)	0.537–1.64	0.826
all no-driver	149	10.7	-	-	-
current *EGFR* del exon 19	22	44.5	0.775 (vs. *EGFR* del exon 19 historic)	0.395–1.52	0.457
historic *EGFR* del exon 19	26	28.6	-	-	-
current *EGFR* exon 21 L858R	12	33.4	0.268 (vs. *EGFR* exon 21 L858R historic)	0.100–0.717	0.0087 **
historic *EGFR* exon 21 L858R	13	13.3	-	-	-
current *EGFR* uncommon	7	9.1	2.04 (vs. *EGFR* uncommon historic)	0.634–6.56	0.232
historic *EGFR* uncommon	7	16.3	-	-	-
***KRAS* subtypes** ^a^	**(Figure 4A–F)**				
*KRAS* G12C	41	19.2	0.869 (vs. nonG12C)	0.507–1.49	0.609
*KRAS* G12A	9	10.0	1.16 (vs. nonG12A)	0.500–2.70	0.728
*KRAS* G12D	7	9.1	3.21 (vs. nonG12D)	0.966–10.7	0.057
*KRAS* G12V	10	37.8	0.864 (vs. nonG12V)	0.386–1.94	0.723
*KRAS* G13X	7	17.2	2.34 (vs. nonG13X)	0.767–7.14	0.135
*KRAS* Q61H	4	25.2	0.913 (vs. nonQ61H)	0.297–2.81	0.874

The “star” (*) cohorts contain only patients fulfilling the major inclusion criteria of the respective phase III studies KEYNOTE-189 and FLAURA. Hazard ratios (HR), confidence intervals (CI), and *p*-values were calculated from the survival proportions of the Kaplan–Meier estimates using the log-rank (Mantel–Cox) test. For significant test results, the significance levels are marked with small asterisks as follows: *: *p* < 0.05; **: *p* < 0.01; ***: *p* < 0.001. ^a^ For six patients with *KRAS* mutation, the subtype was unknown.

## Data Availability

The original data will be made available as a data paper.

## Supplementary Materials

The following Supporting Information can be downloaded at: https://www.mdpi.com/article/10.3390/cancers17071237/s1, Table S1: “Star” (*) Cohorts: Baseline characteristics, treatment, and follow-up of the current and historic cohorts*; Table S2: KRAS-Subtypes: Baseline characteristics, treatment, and follow-up of KRAS-subtype cohorts. Table S3: Comparison with Phase III Trial Results.

## Abbreviations

CPI	checkpoint inhibitor
MDT	multidisciplinary tumor board
NSCLC	non-small cell lung cancer
NGS	next-generation sequencing
OS	overall survival
PCR	polymerase-chain reaction
TKI	tyrosine-kinase inhibitor
TPS	tumor-proportion score
