# Supplementary material for: Trends in Overall Survival in Lung Adenocarcinoma with EGFR Mutation, KRAS Mutation, or No Mutation
Source: Cancers (Basel). 2025 Apr 5;17(7):1237. doi: 10.3390/cancers17071237 (PMC11988053; doi:10.3390/cancers17071237)
Supplement: Supplementary file 1 [file cancers-17-01237-s001.zip › cancers-3548924-supplementary.pdf]

Supplemental Tables:

Table S1: "Star" (*) Cohorts.

	Current *EGFR**	Current no-driver*	Historic *EGFR**	Historic no-driver*
**number**	32	53	29	66
**Date of diagnosis (median, IQR)**	4/2021<br>9/19 to 3/22	3/2020<br>6/17 to 9/22	1/2013<br>2/11 to 9/16	5/2014<br>7/12 to 5/16
**Age (median, range)**	63.2<br>(37.4 –83.3)	65.4<br>(32.3 – 90.8)	66.8<br>(38.8 – 84.8)	65.6<br>(44.6 – 84.8)
Gender				
**Male**	8 (25%)	34 (64%)	4 (14%)	33 (50%)
**Female**	24 (75%)	19 (36%)	25 (86%)	33 (50%)
Performance status				
**ECOG 0**	19 (59%)	12 (23%)	12 (41%)	18 (27%)
**ECOG 1**	13 (41%)	41 (77%)	17 (59%)	48 (73%)
Smoking status		*NA 1*		*NA 1*
**Never-smoker**	15 (47%)	3 (6%)	20 (69%)	12 (18%)
**Ever smoker**	17 (53%)	49 (94%)	9 (31%)	53 (82%)
pack years (median)	*10*	*45*	*10*	*40*
Histology				
**Adenocarcinoma**	31 (97%)	53 (100%)	29 (100%)	66 (100%)
**Adeno-squamous carcinoma**	1 (3%)	0	0	0
TTF1-pos.	*29 (100%)*	*40 (89%)*	*18 (100%)*	33 (72%)
TTF1-neg.	*0*	*5 (11%)*	***0***	13 (28%)
TTF1 NA	*3*	*8*	*11*	*20*
Molecular Subgroups				
***EGFR* del exon 19**	21 (66%)	-	19 (66%)	-
***EGFR* exon 21 L858R**	11 (34%)	-	10 (34%)	-
PD-L1 (TPS)[b]	*NA 5*	*NA 8*	*NA 22*	*NA 36*
**0%**	19 (70%)	22 (49%)	2 (29%)	18 (60%)
**1 – 49%**	6 (22%)	15 (33%)	3 (43%)	7 (23%)
**50 – 100%**	2 (7%)	8 (18%)	2 (29%)	5 (17%)
Stage (UICC 8)				
**Staging included PET-CT**	18 (56%)	36 (68%)	7 (24%)	24 (36%)
**IVA**	11 (34%)	24 (45%)	9 (31%)	21 (32%)
**IVB**	21 (66%)	29 (55%)	20 (69%)	45 (68%)
Treatment included				
**EGFR-TKI**	32 (100%)	0	29 (100%)	0
erlotinib or gefitinib 1L	*1 (3%)*	-	*23 (79%)*	-
afatinib 1L	*1 (3%)*	-	*2 (7%)*	-
osimertinib 1L	*30 (94%)*	-	*4 (14%)*	-
osimertinib ≥2L	*1 (3%)*	-	*5 (17%)*	-
**CPI**	9 (27%)	45 (85%)	2 (7%)	21 (32%)
CPI 1L	*1 (2%)*	*34 (64%)*	*1 (3%)*	*3 (5%)*
CPI 1L pembrolizumab	*1 (2%)*	*32 (60%)*	*0*	*3 (5%)*
CPI ≥2L	*8 (24%)*	*11 (21%)*	*1 (3%)*	*18 (27%)*
**BSC only**	0	3 (6%)	3 (7%)	1 (1%)
Follow-up of living patients (months) [median (range)]	44.7<br>5.0 to 70.8	36.0<br>3.8 to 144.3	92.0<br>1.6 – 151.8	90.4<br>84.9 to 115.6
Deaths	17 (53%)	33 (62%)	24 (83%)	62 (94%)
death due to lung cancer	*17 (53%)*	*25 (47%)*	*19 (66%)*[a]	*60 (91%)*
death due to other causes	*0*	*8 (15%)*	*4 (14%)*	*2 (3%)*

Baseline characteristics, treatment, and follow-up of the current and historic cohorts*.

The “star” (*) cohorts contain only patients fulfilling the major inclusion criteria of the respective phase III studies KEYNOTE-189 and FLAURA. CPI: checkpoint inhibitor; IQR: inter-quartile range; NA: not assessed; NGS: next-generation sequencing; TKI: tyrosine-kinase inhibitor; TPS: tumor proportion score.

[a] One cause of death was unknown.

Table S2: *KRAS*-Subtypes

	KRAS	*KRAS G12C*	*KRAS G12A*	*KRAS G12D*	*KRAS G12V*	*KRAS G13X*	*KRAS Q61H*
**Number**	90	41	9	7	10	7	4
**Median date of diagnosis**	8/2020	7/2021	8/2019	5/2021	2/2021	3/2020	9/2019
**Age (median, range)**	65 (39-87)	68 (39-82)	64 (44-79)	59 (45-87)	74 (51-87)	63 (48-69)	58 (56-67)
Sex							
**Male**	46 (51%)	21 (51%)	4 (44%)	4 (57%)	6 (60%)	3 (43%)	2 (50%)
**Female**	44 (49%)	20 (49%)	5 (56%)	3 (43%)	4 (40%)	4 (57%)	2 (50%)
Performance status							
**ECOG 0-1**	79 (88%)	37 (90%)	6 (67%)	5 (71%)	10 (100%)	6 (86%)	4 (100%)
**ECOG 2-3**	11 (12%)	4 (10%)	3 (33%)	2 (29%)	0	1 (14%)	0
Smoking status	*NA 1*			*NA 1*			
**Never-smoker**	5 (6%)	19 (46%)	1 11%)	1 (17%)	3 (30%)	0	0
**Ever-smoker**	84 (94%)	22 (54%)	8 (89%)	5 (83%)	7 (70%)	7 (100%)	4 (100%)
PD-L1 (TPS)[d]	*NA 12*	*NA 6*	*NA 1*	*NA 1*	*NA 2*		*NA 1*
**0%**	23 (29%)	23 (66%)	1 (13%)	4 (67%)	1 (13%)	3 (43%)	0
**1 – 49%**	23 (29%)	9 (26%)	2 (25%)	1 (17%)	4 (50%)	1 (14%)	0
**50 – 100%**	32 (41%)	3 (9%)	5 (63%)	1 (17%)	3 (38%)	3 (43%)	3 (100%)
Stage (UICC 8)							
**IVA**	36 (40%)	15 (37%)	5 (56%)	2 (29%)	4 (40%)	2 (29%)	0
**IVB**	54 (60%)	26 (63%)	4 (44%)	5 (71%)	6 (60%)	5 (71%)	4 (100%)
Treatment included							
**CPI**	79 (88%)	39 (95%)	7 (78%)	6 (86%)	9 (90%)	7 (100%)	4 (100%)
Deaths	58 (64%)	23 (56%)	6 (67%)	6 (86%)	6 (60%)	6 (86%)	3 (75%)
Death due to lung cancer	*48 (53%)*[a]	*18 (44%)*[b]	*4 (44%)*[b]	*6 (86%)*	*5 (50%)*	*5 (71%)*	*3 (75%)*
Death due to other causes	*9 (10%)*	*4 (10%)*	*1 (11%)*	*0*	*1 (10%)*	*1 (14%)*	*0*

Baseline characteristics, treatment, and follow-up of *KRAS*-subtype cohorts.

CPI: checkpoint inhibitor; NA: not assessed.

The subtype was unknown in 6 *KRAS* patients. Subtypes with n≥4 were analyzed. *KRAS* G12F (n=3), *KRAS* G12R (n=1), and *KRAS* G12S (n=2) were not analyzed.

[a] Unknown cause of death in 2 patients. [b] Unknown cause of death in 1 patient.

Table S3: Comparison with Phase III Trial Results

	Current no-driver* (n=53)	Historic no-driver* (n=66)	KEYNOTE-189 pembrolizumab+chemotherapy	KEYNOTE-189 chemotherapy
**CPI 1L**	45 (85%)	22 (33%)	100%	100%
CPI 1L including pembrolizumab	*34 (64%)*	*3 (4%)*	*100%*	*0*
**Median OS**	18.7 months	10.3 months	22.0 months	10.6 months
**12 months OS**	63%	44%	70%	48%
**24 months OS**	38%	27%	46%	27%
**36 months OS**	30%	15%	32%	18%
**HR (CI)**	0.60 (vs. historic no-driver*) (CI 0.40 to 0.89)		0.49 (vs. chemotherapy) (CI 0.38 to 0.64)	

Table S3A.

	Current *EGFR** (n=32)	Historic *EGFR** (n=29)	FLAURA Osimertinib	FLAURA EGFR-TKI comparator (gefitinib or erlotinib)
**EGFR-TKI 1L**	32 (100%)	29 (100%)	100%	100%
Gefitinib or erlotinib 1L	*1 (3%)*	*23 (79%)*	*0*	*100%*
Afatinib 1L	*1 (3%)*	*2 (7%)*	*0*	*0*
**Osimertinib 1L**	30 (94%)	4 (14%)	100%	0
**Osimertinib 2L**	1 (3%)	5 (17%)	-	NA
**Median OS**	44.5 months	29.6 months	38.6 months	31.8 months
**12 months OS**	93%	82%	89%	83%
**24 months OS**	79%	61%	74%	59%
**36 months OS**	63%	39%	54%	44%
**HR (CI)**	0.68 (vs. EGFR* historic) (CI 0.36 to 1.28)		0.80 (vs. EGFR-TKI comparator) (CI 0.64 to 1.00)	

Table S3B.

S3A. Overall survival (OS) of current and historic no-driver patients and performance status ECOG 0-1. ECOG ≥ 2 patients were excluded to get cohorts matching the KEYNOTE-189 trial population.[23]

S3B. Overall survival (OS) of current and historic *EGFR* patients with common mutations (del exon 19 or exon 21 L858R) and performance status ECOG 0-1. Uncommon mutations and ECOG ≥ 2 patients were excluded to get cohorts matching the FLAURA trial population.[44]

Hazard ratios (HR), confidence intervals (CI) and p-values were calculated from the survival proportions of the Kaplan-Meier estimates using the log-rank (Mantel-Cox) test.
